# Efficacy of a Novel Rotary System in Reduction of Intracanal Bacteria: An *in Vitro* Study

**DOI:** 10.7508/iej.2016.03.014

**Published:** 2016-05-01

**Authors:** Maryam Vossoghi, Mitra Vossoghi, Shahriar Shahriari, Farhad Faramarzi, Rasoul Yousefi Mashouf, Maryam Farhadian

**Affiliations:** a *Prosthodontics Department, Kerman University of Medical Sciences, Kerman, Iran; *; b * Endodontic Department, Faculty of Dentistry, Ilam University of Medical Sciences, Ilam, Iran; *; c * Endodontic Department, Faculty of Dentistry, Hamedan University of Medical Sciences, Hamedan, Iran; *; d * Department of Medical Microbiology, Hamedan University of Medical Sciences, Hamedan, Iran; *; e *Department of Epidemiology and Biostatistics, School of Public Health, Hamadan University of Medical Science, Hamadan, Iran*

**Keywords:** *Enterococcus faecalis*, iRace, OneShape, ProTaper

## Abstract

**Introduction:**

This *in vitro* study aimed to assess the effectiveness of a single-file rotary system (OneShape) in reduction of intracanal bacteria.

**Methods and Materials:**

Eighty one single-rooted mandibular first premolars with single canals were used. Six samples were selected as aseptic control group. Seventy five remaining specimens were infected by *Enterococcus faecalis* and incubated for 72 h at 37^°^C. Then they were randomly divided into three groups (*n*=25). In each group, cleaning and shaping procedures were done using either two conventional rotary systems (ProTaper and iRace), or the single-file system (OneShape). Microbial samples from the intracanal environment were taken by paper points in two steps, before and after instrumentation. Then, they were diluted and plated in blood agar. In order to compare bacterial reduction and turbidity, the Kruskal-Wallis test was used followed by the Chi-Square and Mann-Whitney tests for pairwise comparison. The level of significance was set at 0.05.

**Results:**

The control group didn’t show any bacterial growth. The pre- and post-instrumentation samples were significantly different between three groups (*P*=0.02). Hence, there was no significant differences between turbidity of samples (*P>0.05)*.

**Conclusion:**

OneShape system is efficient in bacterial reduction. In this regard ProTaper is the most effective system in intracanal bacterial reduction followed by iRaCe and OneShape, respectively.

## Introduction

Microorganisms play an important role in establishment of pulp and periapical diseases. So it is essential to eradicate them as etiologic factors, during endodontic treatment [1]. Reducing bacteria from infected root canals needs a combined use of antimicrobial irrigants, mechanical instrumentation and intracanal antibacterial medicaments. Among these mechanical instrumentation is fundamental [2].

Bacteria can penetrate deeply into the dentinal tubules especially at coronal and middle zones of root canals. So it is possible that files with greater tapers could reduce bacteria more than smaller taper files [1]. *Enterococcus faecalis* (*E*. *faecalis*) is highly resistant to antimicrobial irrigants and it can adhere, grow and penetrate into dentinal tubules and resist against host defense. As a result this cocci can be the reason for persistent endodontic lesions [3].

In recent years, the advances in development of endodontic instruments, has increased the prognosis of endodontic treatments [4]. Introduction of NiTi engine-driven systems has opened a new era in endodontic treatment. ProTaper (Dentsply Maillefer, Ballaigues, Switzerland) and iRace (FKG Dentaire, La-Chaux-de Fonds, Switzerland) are two popular rotary systems with multiple files. There are many studies which have evaluated their ability in reduction of intracanal bacteria [1, 3-7]. Recently new engine-driven systems with the ability to clean and shape the canals with single files, are presented for the clinician’s comfort and decreased chair time [8]. One of these new single file systems is OneShape (Micromega, Besancon, France) which is used in continuous clockwise rotation for a quick root canal preparation [9]. OneShape (25/0.06) has asymmetrical cross-section along the entire blade (which limits the risks of instrument breakage due to the accumulation of stresses on the file), variable cross-section and longer pitch. These properties cause reduction of the preparation time, efficient cleaning, decrease in the bacterial charge similar to that of traditional instruments and lower quantity of apically extruded debris [10]. 

Rare researches have evaluated the efficacy of single-file systems in removing intracanal bacteria as the main purpose of mechanical instrumentation [11]. Therefore the aim of the present *in vitro *study was to compare the bacterial-reduction ability of this single-file system with two popular multiple-instrument rotary systems during root canal treatment.

## Materials and Methods

The study protocol was approved by Research Technology Deputy of Hamedan University of Medical Sciences. Eighty one extracted single-rooted mandibular first premolars with single canals were collected. After disinfecting the teeth with 5.25% NaOCl (Darugar, Tolipers, Iran) for 1 h, the teeth were stored in sterilized physiological solution until use. The specimens were decoronated by diamond disk and the root lengths were standardized to 15±2 mm. The working length was determined by a #15 K-file (Dentsply, Maillefer, Ballaigues, Switzerland) after reducing 1 mm from its length upon emergence through the apical foramen. For removing the smear layer, the root canals were irrigated with 17% EDTA (RC-Prep, Premier Dental Products, Norristown, PA, USA) for 3 min and finally washed with 5 mL distilled water. The apical foramen was sealed with cyanoacrylate resin and the root surface was covered with two layers of nail polish. Then the teeth were placed in micropipette and fixed with acrylic resin. All specimens were autoclaved at 121^°^C and 15 psi pressure for 15 min.

Six specimens were filled by sterile brain-heart infusion (BHI) broth (Difco, Baltimore, MD, USA). By using #10 µL samplers, a suspension of *E. faecalis*, standardized to #4 McFarland scale, were inoculated into the remaining 75 samples. All specimens were then incubated at 37^°^C for 72 h. Then, they were randomly divided into 3 groups (*n*=25). First, the root canals were filled with sterile BHI broth and initial samples were collected by 3 sterilized #15 paper points (Gapadent, Hamburg Germany) inserted for 1 min. Next, the paper points were stored in tubes of containing 1 mL sterile BHI broth for 10 min. Then serial bacterial dilutions were prepared. For bacterial count in colony-forming units (CFUs)/mL, 2, 5 and 10 µL dilutions were plated in blood agar culture medium and plates were incubated at 37^°^C for 48 h.

Contaminated specimens were instrumented by dividing them into the following 3 groups. In group 1 instrumentation with ProTaper system (Dentsply Maillefer, Ballaigues, Switzerland) was first initiated with brushing movements of SX and then followed by S2, S1, S2, F1, F2 and F3 installed on a gear reduction handpiece (Sirona Dental Systems GmbH, Bensheim, Germany) powered by a torque-controlled motor (Silver; VDW GmbH, Munich, Germany) adjusted in speed and torque of 300 rpm and 3 N/cm, respectively [4].

In group 2, iRaCe files (15/0.06, 25/0.04 and 30/0.04) (FKG Dentaire, La-Chaux-de Fonds, Switzerland) were used according to manufacturer’s instructions with gentle in and out strokes. If 15/0.06 file faced resistance, additional instruments (20/0.02 and 25/0.02) were used. Optimum speed and torque were 600 rpm and 1.5 N/cm, respectively [6].

In group 3, 25/0.06 OneShape file (Micro Méga, Besançon, France) was used with Endo IT motor in continuous motion at 400 rpm and a torque of 4 N/cm. According to the manufacturer the file was used in three in-and-out motions before irrigation. Preparation of the canals continued until reaching the working length [8, 9].

During instrumentation, all root canals were irrigated thoroughly with 5 mL solution of 5.25% NaOCl. The specimens of control group were not contaminated, nor instrumented. After preparing the root canals, they were filled with 5 mL distilled water and incubated at 37^°^C for 24 h for determining the presence or absence of *E. faecalis*. The procedure was done in the laminar flow hood. Sterilized #30 paper points were inserted into root canals for 1 min and similar process of initial sampling was repeated. Additionally, to evaluate the turbidity the tubes were incubated at 37^°^C for 24 h. If the tubes were turbid, they were cultured by streak method to confirm the growth of *E. faecalis*. Statistical analysis was performed using descriptive analysis and Kruskal-Wallis, Chi-Square and Mann-Whitney tests. The level of significance was set at 0.05.

**Table 1.** Number (percentage) of tube turbidity after 24 h

**Group turbidity **	**OneShape**	**ProTaper**	**iRaCe**
**No**	13 (52)	16 (64)	15 (60)
**Yes**	12 (48)	9 (36)	10 (40)

**Table 2:** Mean (SD) of bacterial reduction and counts (CFU) of *E. faecalis* before and after instrumentation

**Group (N) **	**Pre-instrumentation**	**Post-instrumentation**	**Bacterial reduction (%)**
**iRaCe (25)**	7.2×10^4^ (3.6×10^4^)	4 (20)	99.99±0.02
**ProTaper (25)**	7.7×10^4^ (3.7×10^4^)	0 (0)	100±0
**OneShape (25)**	7.3×10^4^ (3.6×10^4^)	9.4×10^2^ (38.9×10^2^)	97.92±5.77

## Results

The control group did not show any bacterial growth and it revealed aseptic condition during procedure.

Based on turbidity of tubes, there were no significant differences between groups (*P*=0.68). Table 1 shows the percentage of turbidity in each group after a 24-h incubation. Mean values of CFU before and after instrumentation and percentage of bacterial reduction are shown in Table 2. 

According to the Kruskal-Wallis analysis, there was a significant difference in bacteria reduction after instrumentation between three groups (*P*=0.02). Pairwise comparison of ProTaper-iRaCe and iRaCe-OneShape, using the Mann-Whitney test, showed no significant differences (*P*=0.31 and *P*=0.07, respectively). But there was a significant difference between ProTaper and OneShape (*P*=0.02).

## Discussion

Necrotic pulp and infected dentin play an important role in producing apical lesions. The aim of endodontic treatment is to reduce intracanal bacteria and their byproducts [1, 11]. The present *in vitro *study compared the efficacy of three different engine-driven endodontic systems in removal of intracanal bacteria from the root canal system. The methodology used in this study was chemomechanical preparation similar to Nakamura *et al.* [12], Ferrer-Luque *et al.* [13] and Coldero *et al.* [14]. Due to the importance of bacteria removal in a successful endodontic treatment, bacteriological assessment was used. Due to the clinical importance, high resistance to antibacterial solutions and deep penetration into the dentinal tubules, *E. faecalis* was chosen as the bacteriologic marker. It has also been traced in persistent endodontic lesions [15-17].

Among dental groups, single-rooted teeth with single canals were selected as done by Matos Neto *et al*. [3], Eskandarinejhad *et al.* [18] and Nazari Moghaddam *et al.* [19]. Other roots have variations that impedes cleaning all root canal spaces and may cause error in the results. 

The amount of bacterial reduction for the first two rotary systems were 100% and 99.99% for ProTaper and iRaCe, respectively. While for OneShape system this reduction was 97.92%. Although the index for all groups were greater than 95% [8, 12, 20], but there was a significant difference between these three groups (*P*=0.02). These results are similar to other studies such as the study by Nabeshima *et al.* [8] who reported 96.5% bacterial reduction for OneShape. Also Martinho *et al.* [11] reported 99.85% reduction for ProTaper and this criterion was reported 98.6% for RaCe rotary system by Zarabian *et al.* [5]. It should be noted that Martinho *et al. *[11]*,* irrigated the root canals with 2.5% NaOCl during instrumentation the same as the present study. However, Matos *et al. *[3], reported 75.61% bacterial reduction and used single rooted human canines. Machado *et al. *[4]*,* reported 81.94% bacterial reduction and used distobuccal canals of upper molars for ProTaper rotary system. They irrigated canals with distilled water during instrumentation.

In the present study all specimens were irrigated with 5.25% NaOCl during instrumentation according to OneShape protocol. Due to the turbidity of some tubes which are indicated in Table 1, we concluded that 100% bacterial reduction doesn’t indicate the absence of bacteria; rather, it shows a very low amount of bacteria that cannot be detected by culture methods.

## Conclusion

The results of this study show that the OneShape system can significantly reduce CFU in the infected root canals similar to ProTaper and iRaCe rotary systems. However, ProTaper system is more effective which may be due to greater taper of ProTaper files.
